# Supplementary figures and images for: A novel human pluripotent stem cell gene activation system identifies IGFBP2 as a mediator in the production of haematopoietic progenitors in vitro
Source: eLife. 2024 Dec 23;13:RP94884. doi: 10.7554/eLife.94884 (PMC11666236; doi:10.7554/eLife.94884)

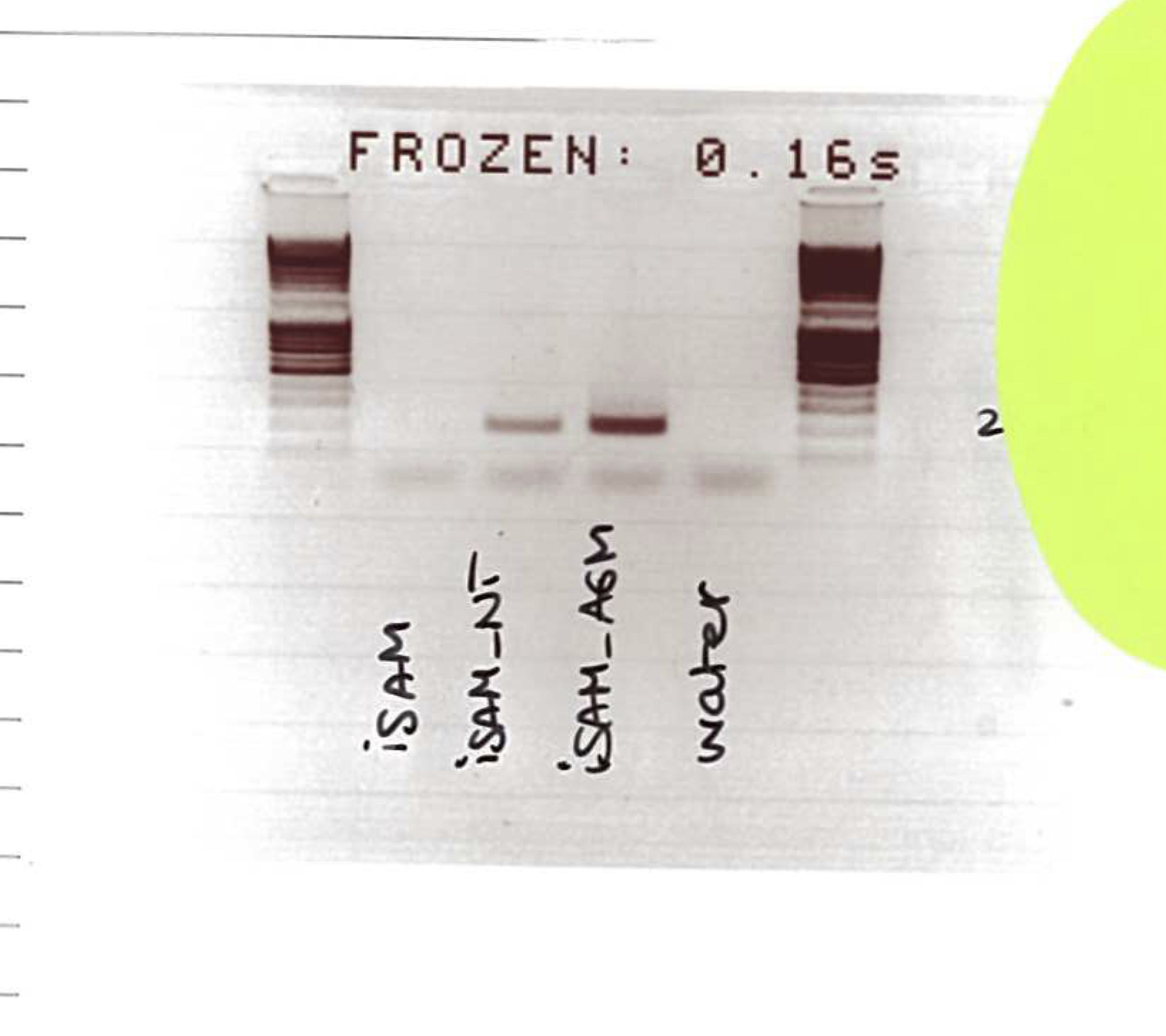

Supplement: Figure 2—figure supplement 2—source data 2. [file elife-94884-fig2-figsupp2-data2.zip › Figure 2 - figure supplement 2 - data source 2/FIgure 2 - figure supplement 2 - source data 3.tiff]

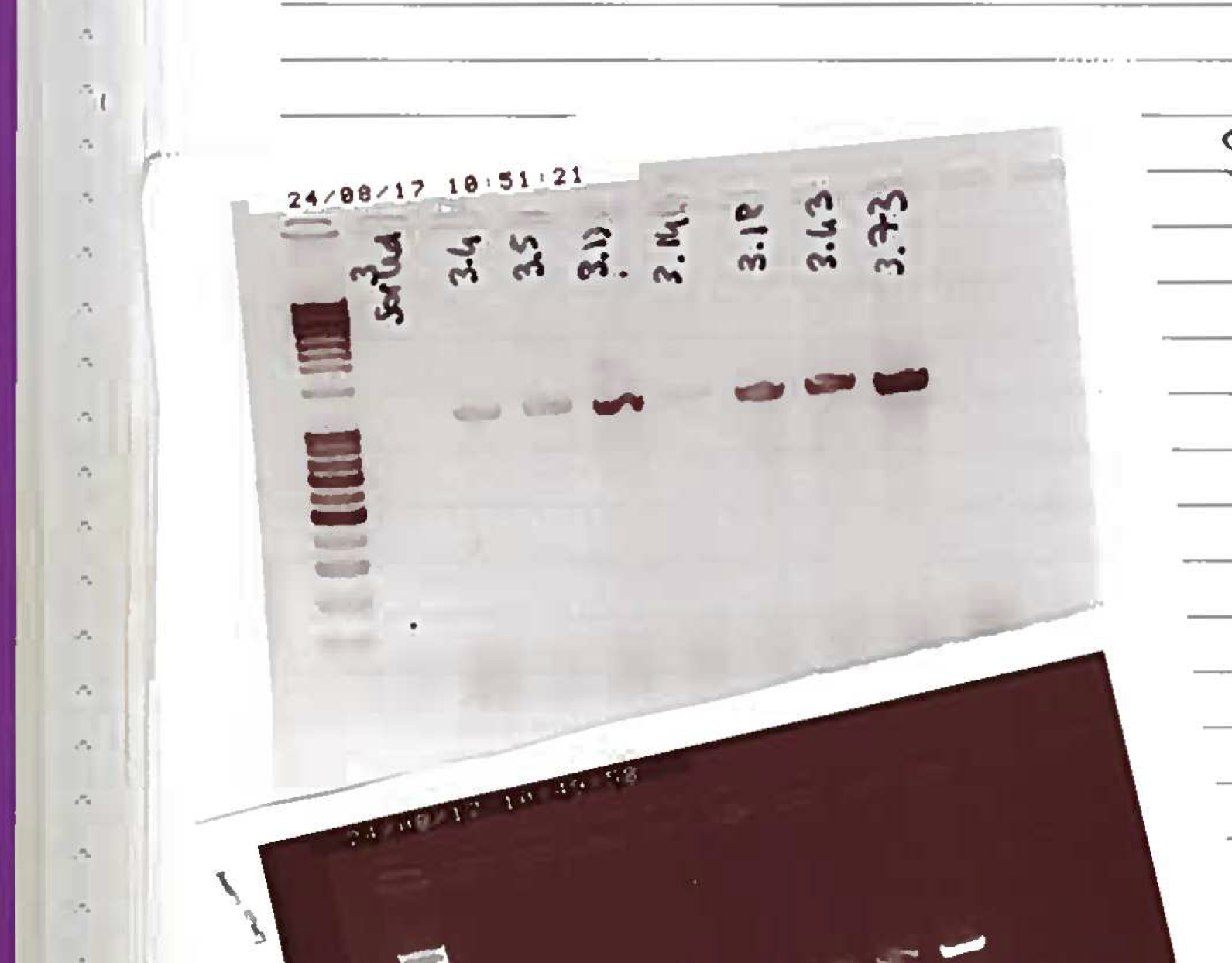

Supplement: Figure 2—figure supplement 2—source data 2. [file elife-94884-fig2-figsupp2-data2.zip › Figure 2 - figure supplement 2 - data source 2/FIgure 2 - figure supplement 2 - source data 1.tiff]

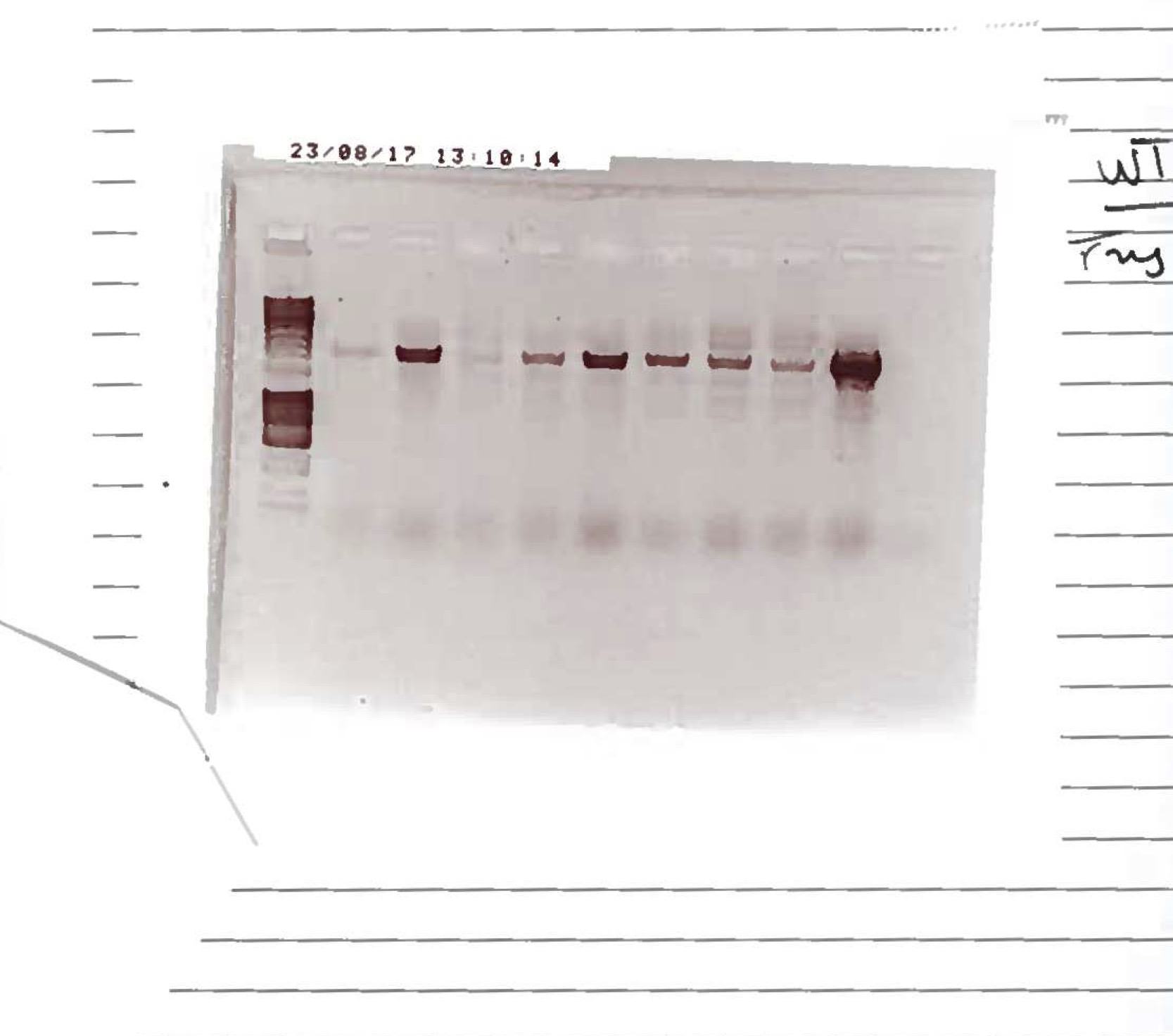

Supplement: Figure 2—figure supplement 2—source data 2. [file elife-94884-fig2-figsupp2-data2.zip › Figure 2 - figure supplement 2 - data source 2/FIgure 2 - figure supplement 2 - source data 2.jpg]

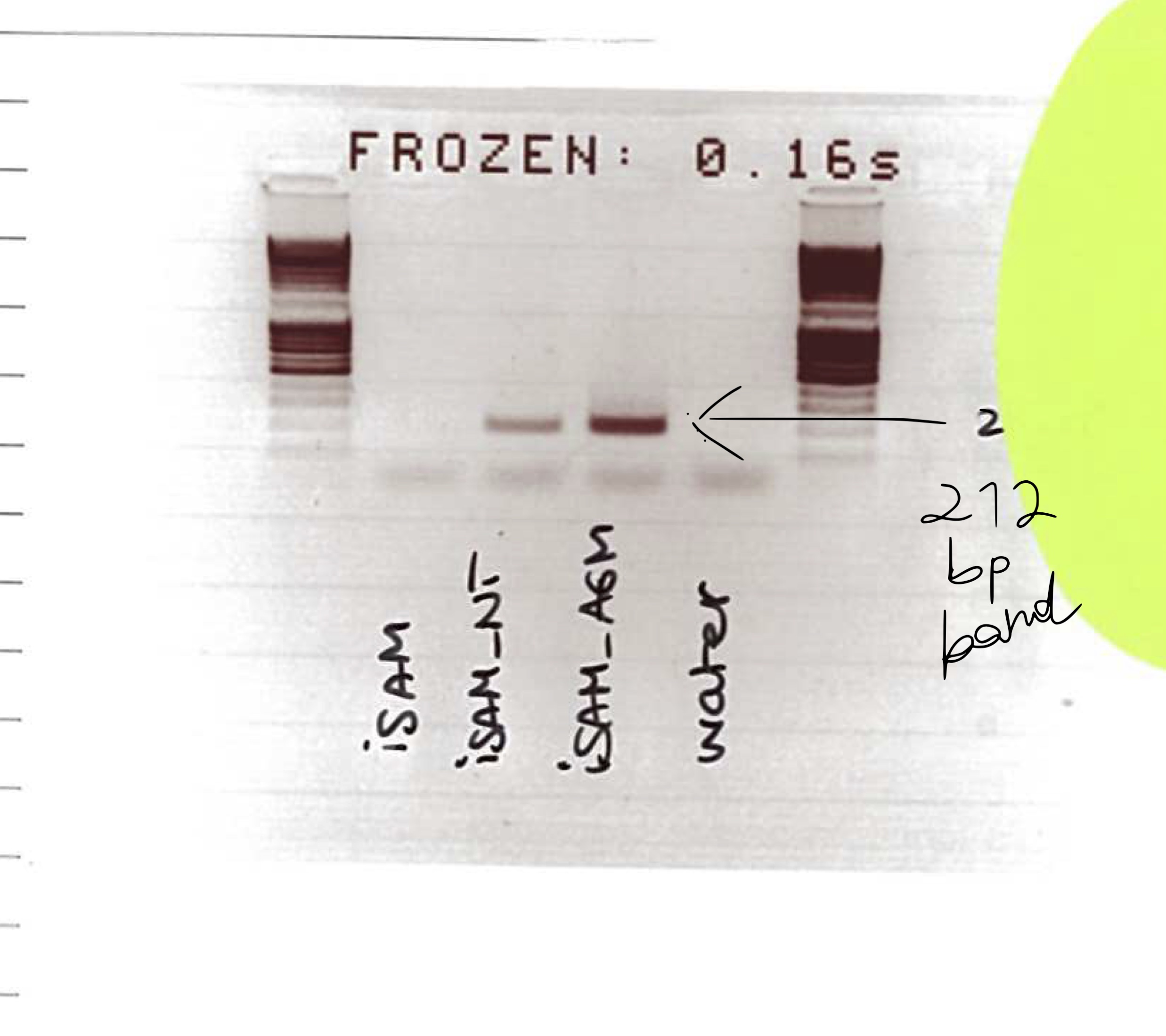

Supplement: Figure 2—figure supplement 2—source data 3. [file elife-94884-fig2-figsupp2-data3.zip › Figure 2 - figure supplement 2 - source data 2 labelled/FIgure 2 - figure supplement 2 - source data 3 LABELLED.jpg]

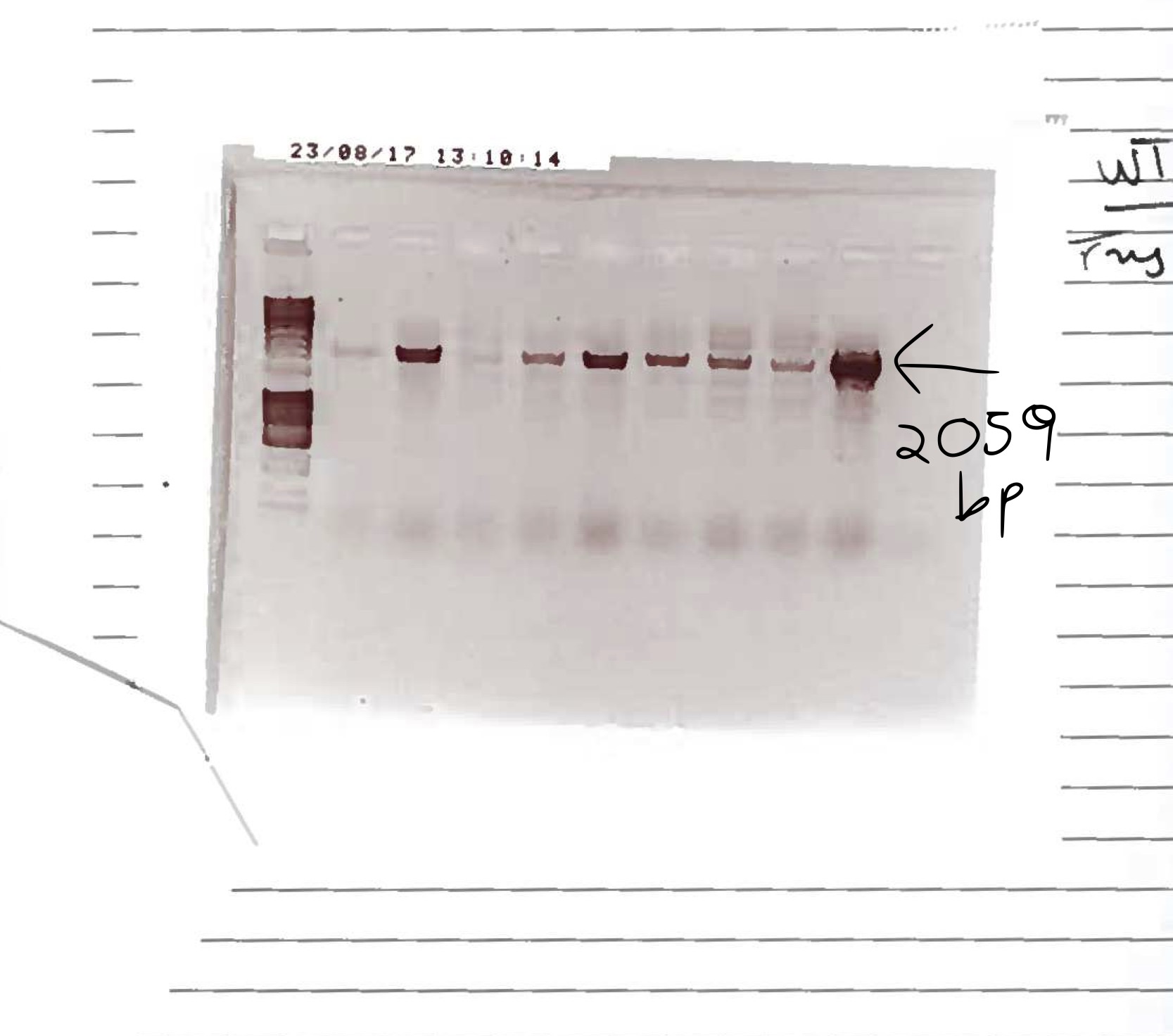

Supplement: Figure 2—figure supplement 2—source data 3. [file elife-94884-fig2-figsupp2-data3.zip › Figure 2 - figure supplement 2 - source data 2 labelled/FIgure 2 - figure supplement 2 - source data 2 LABELLED.jpg]

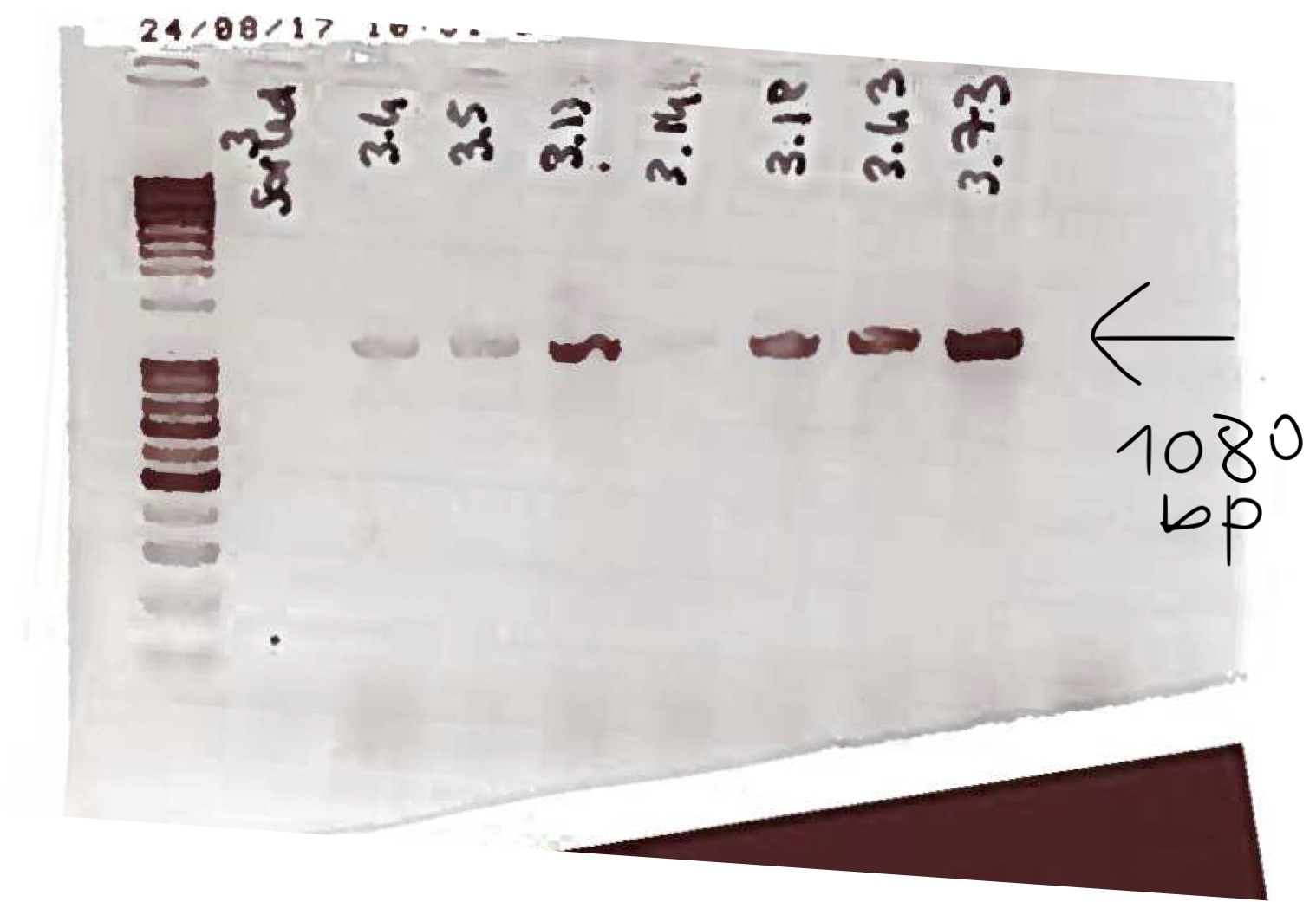

Supplement: Figure 2—figure supplement 2—source data 3. [file elife-94884-fig2-figsupp2-data3.zip › Figure 2 - figure supplement 2 - source data 2 labelled/FIgure 2 - figure supplement 2 - source data 1 LABELLED.jpg]
